# Paired acoustic recordings and point count surveys reveal Clark's nutcracker and whitebark pine associations across Glacier National Park

**DOI:** 10.1002/ece3.10867

**Published:** 2024-01-24

**Authors:** Vladimir Kovalenko, Jeffrey W. Doser, Lisa J. Bate, Diana L. Six

**Affiliations:** ^1^ Department of Ecosystem and Conservation Sciences University of Montana Missoula Montana USA; ^2^ Science Center Glacier National Park West Glacier Montana USA; ^3^ Department of Integrative Biology, Ecology, Evolution and Behavior Program Michigan State University East Lansing Michigan USA

**Keywords:** acoustic monitoring, Clark's nutcracker, habitat use, mutualist, whitebark pine

## Abstract

Global declines in tree populations have led to dramatic shifts in forest ecosystem composition, biodiversity, and functioning. These changes have consequences for both forest plant and wildlife communities, particularly when declining species are involved in coevolved mutualisms. Whitebark pine (*Pinus albicaulis*) is a declining keystone species in western North American high‐elevation ecosystems and an obligate mutualist of Clark's nutcracker (*Nucifraga columbiana*), an avian seed predator and disperser. By leveraging traditional point count surveys and passive acoustic monitoring, we investigated how stand characteristics of whitebark pine in a protected area (Glacier National Park, Montana, USA) influenced occupancy and vocal activity patterns in Clark's nutcracker. Using Bayesian spatial occupancy models and generalized linear mixed models, we found that habitat use of Clark's nutcracker was primarily supported by greater cone density and increasing diameter of live whitebark pine. Additionally, we demonstrated the value of performing parallel analyses with traditional point count surveys and passive acoustic monitoring to provide multiple lines of evidence for relationships between Clark's nutcracker and whitebark pine forest characteristics. Our findings allow managers to gauge the whitebark pine conditions important for retaining high nutcracker visitation and prioritize management efforts in whitebark pine ecosystems with low nutcracker visitation.

## Graphical abstract

Whitebark pine (*Pinus albicaulis*) is a rapidly declining keystone species in high‐elevation ecosystems and an obligate mutualist of Clark's Nutcracker (*Nucifraga columbiana*), an avian seed predator and disperser. Using passive acoustic monitoring, point count surveys, and Bayesian occupancy models, we found that habitat use of Clark's Nutcracker is primarily influenced by cone production and increasing diameter of live whitebark pine, emphasizing the importance of restoration strategies that work to protect existing large cone‐bearing whitebark pines.

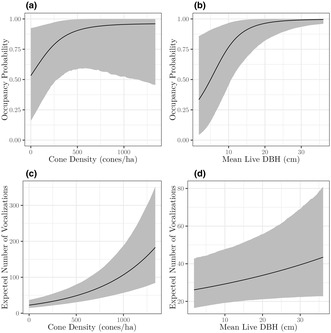

## Анотація

Глобальні зменшення популяцій дерев призвело до різких змін у складі лісових екосистем, біорізноманіття та функціонування. Ці зміни мають наслідки як для лісових рослин, також для диких тварин, особливо коли види, що скорочуються, залучені до коеволюційних мутуалізмів. Кількість білокорої сосни (*Pinus albicaulis*) скорочується у високогірних екосистемах західної Північної Америки і є обов'язковим мутуалістом горіхівки Кларка (*Nucifraga columbiana*), пташиного хижака, який розповсюджує насіння. Доповнюючи традиційний метод підрахування з результатами пасивного акустичного моніторингу, ми досліджували як характеристики ділянок білокорої сосни на охоронюваній території (Національний парк Глейшер, штат Монтана, США) впливають на розміщення та голосової активності горіхівки Кларка. Використовуючи Байєсівські моделі просторового розміщення та узагальнені лінійні змішані моделі, ми виявили, що середовище існування горіхівки Кларка в першу чергу підтримується більшою кількістю шишок а також більшим діаметром живої білокорої сосни. Наші висновки дозволяють менеджерам ресурсів оцінювати стан білокорої сосни, необхідний для збереження високого рівня знаходження горіхівки і визначити пріоритетність зусиль для відновлення їх в районах, де вона низька. Наша структура також надає менеджерам протокол відбору проб, який полегшує довгостроковий моніторинг екосистеми білокорої сосни.

## 1 INTRODUCTION

Forest ecosystems are undergoing substantial shifts in composition and structure across macroscales (Allen et al., 2010; Stanke et al., 2021). These ecosystem‐level responses are largely driven by increasing tree mortality due to a variety of interacting processes such as invasive species (Anagnostakis, 1987), climate change (Yuan et al., 2019), and an increasing prevalence of severe wildfires (Fried et al., 2004; Westerling, 2016). Such shifts in tree population dynamics can have large impacts on ecosystem functioning and services (Tomback et al., 2016) including effects on wildlife that rely on forest stands for breeding and foraging. Many bird species play key roles in forest systems via effects of their foraging and seed dispersal activities which ultimately influence many ecosystem functions (Wenny et al., 2011; Whelan et al., 2008). Because forest birds simultaneously rely on forest ecosystems for nesting substrates, food resources, and shelter, shifts in tree population dynamics can also have considerable effects on forest bird populations (Bonter & Harvey, 2008; Bregman et al., 2014; Ralston et al., 2015).

Whitebark pine (*Pinus albicaulis*) is a keystone species of high‐elevation ecosystems in the western U.S. and Canada, providing a suite of ecological functions including snowpack retention, early successional community establishment, and food for many wildlife species (Ellison et al., 2005; Tomback et al., 2001, 2016). Whitebark pine is in steep decline in portions of its range and was listed as threatened by the U.S. Fish and Wildlife Service in 2023 and endangered by the Canadian government in 2012 (Government of Canada, 2012; U.S. Fish & Wildlife Service, 2022). Several factors are contributing to the decline of whitebark pine. In the more mesic portions of its range, the main threat is currently an introduced pathogen, *Cronartium ribicola*, that causes the often fatal disease white pine blister rust (Arno, 1986; Six et al., 2018; Tomback & Achuff, 2010). As a result, a federally‐mandated recovery plan is currently being developed for the tree in the U.S., with a similar plan in Canada (Environment and Climate Change Canada, 2017; Tomback & Sprague, 2022; U.S. Fish & Wildlife Service, 2022). However, a lack of information on many aspects of whitebark pine ecology hinders the development of conservation and restoration of affected ecosystems.

One area of uncertainty is how well the tree will be able to regenerate in areas where its mortality has been high. Natural regeneration of whitebark pine is reliant on a mutualistic relationship with Clark's nutcracker (*Nucifraga columbiana*). Clark's nutcracker is a jay‐sized corvid and the only member of the genus *Nucifraga* native to North America (Tomback, 1978). It primarily inhabits the montane forests of the western United States and Canada where it feeds on conifer seeds and insects (Giuntoli & Mewaldt, 1978). It disperses the seed of several conifers, including whitebark pine, via caching for later recovery for food, including to feed nestlings that hatch prior to snowmelt (Mattes, 1984). Seeds that are not recovered may germinate to produce new trees (Lorenz & Sullivan, 2009). Unlike other tree seeds used by nutcrackers, whose seeds are wind dispersed, whitebark pine produces closed cones that require opening by nutcrackers to free the seeds. The birds then place the seeds into a sublingual pouch and fly some distance to cache the seeds in soil. Evidence suggests that regeneration of whitebark pine is completely dependent on nutcracker seed caching (Hutchins & Lanner, 1982; Lorenz, 2008; Tomback, 1982).

Declines of whitebark pine may be altering the mutualism between the bird and the tree (Barringer et al., 2012; McKinney et al., 2009; McKinney & Tomback, 2007; Ray et al., 2020; Schaming, 2015) leading to poor or no regeneration of whitebark pine and negative effects on Clark's nutcracker populations. In areas where whitebark pine has declined substantially, nutcrackers may consume all or most available seeds and may reduce or cease visitation to, and caching in, stands with low cone production (McKinney & Tomback, 2007; Siepielski & Benkman, 2007). Indeed, there is evidence that when cone production falls below a threshold, nutcracker visitation becomes more variable and less likely (Barringer et al., 2012; McKinney et al., 2009). McKinney et al. (2009) estimated a cone production threshold of about 700 cones per ha was necessary to maintain a nutcracker occurrence probability of >40%, which corresponds to a live whitebark pine basal area of more than 5 m^2^ per ha. Maier (2012) observed that nutcrackers in Glacier National Park (GNP), where whitebark pine mortality has been high, continued to visit all cone‐bearing whitebark stands, although it was unclear whether birds were dispersing or immediately consuming seeds (Kovalenko, 2023; Maier, 2012). Barringer et al. (2012) found evidence for a threshold effect similar to that found by McKinney et al. (2009), but concluded that the proportion of living whitebark pine rather than cone density was the best predictor of nutcracker occurrence, with increasing uncertainty in visitation with a live whitebark pine basal area of 2 m^2^ per ha or lower (Barringer et al., 2012). In another study, Schaming and Sutherland (2020) found the presence of cone‐bearing whitebark pines to be a more important predictor of nutcracker occurrence than cone density and that the variability in nutcracker occurrence increases at higher cone densities. These results together indicate a need for additional research into the relationship between Clark's nutcracker and whitebark pine in the Northern Rockies and elsewhere throughout their range.

Nutcracker visitation and caching is not only important for whitebark pine regeneration but also potentially for the viability of nutcracker populations. The bird is often considered a facultative mutualist with whitebark pine because it also forages for seed on other conifers over the year. However, Schaming (2015) found a lack of breeding by nutcrackers in years with whitebark cone crop failures, suggesting strong fitness effects on the bird that may indicate more dependency than previously recognized.

High uncertainty in the drivers of nutcracker habitat use and estimates of occurrence in past studies may be a product of Clark's nutcrackers' highly irruptive migration habits, large‐scale movements (i.e., 2–3 km in a single flight) between harvest and caching sites, and limitations in traditional sampling methods (i.e., point count and transect surveys) to detect highly mobile species like Clark's nutcracker (Barringer et al., 2012; DeSante & Saracco, 2021; McKinney et al., 2009; Sauer et al., 2014; Schaming & Sutherland, 2020; Tomback et al., 2001). The use of automated acoustic recording devices may augment traditional survey methods (Cole et al., 2022; Doser et al., 2021) and increase the potential to detect the target species, especially when it is rare (Sugai et al., 2019). Unlike traditional surveys that rely on direct observations, acoustic recording units record animal vocalizations, providing a non‐invasive and efficient way to collect data on their presence and activity rates (Browning et al., 2017). Acoustic monitoring may be particularly valuable for monitoring highly mobile species like Clark's nutcracker, which produce loud and unique vocalizations.

Here we modeled occupancy and vocalization activity using point count survey and acoustic monitoring data, respectively, to assess the drivers of Clark's nutcracker occupancy and activity in whitebark pine forest ecosystems in Glacier National Park from 2020 to 2022. By using two independent modeling approaches in parallel, we were able to obtain a more informed understanding of which whitebark pine characteristics most strongly relate to nutcracker occupancy and activity. Specifically, we asked the following questions:

1. Do the whitebark pine stand characteristics of cone density, mean tree diameter, basal area, and proportion of individuals infected with white pine blister rust influence nutcracker occupancy and vocalization activity rates?
2. Did nutcracker occupancy and mean daily vocalization activity rates vary over the three‐year period of our study?
3. How do results from traditional point count surveys compare to those from acoustic recording units?

## 2 METHODS

### 2.1 Study area

**FIGURE 1:**
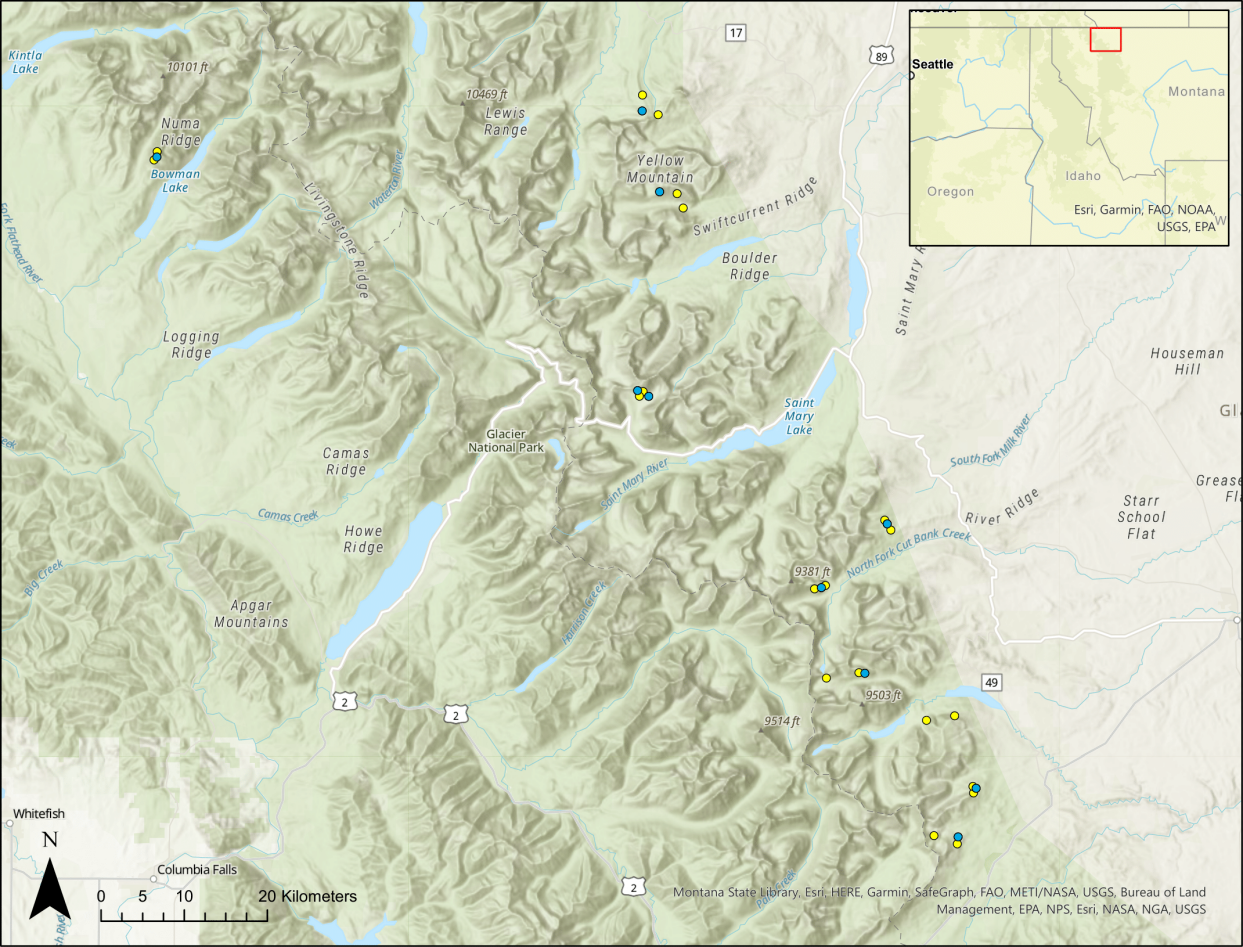
Study area in Glacier National Park, Montana, USA. Points indicate the locations of *Nucifraga columbiana* point count stations included in the study. Yellow points indicate locations of automated recording units, while blue points had point count surveys only.

GNP comprises approximately 4100 km^2^ of the Northern Divide Ecosystem in northwestern Montana, USA (Figure 1). The park encompasses numerous ecosystems and ecotones, with an elevation gradient ranging from 1000 m to over 3000 m (GNP webpage, https://www.nps.gov/glac/learn/nature/index.htm). Within GNP, Clark's nutcracker forages upon the whitebark pine cone crop beginning in mid‐ to late‐July through late September, depending on cone availability during this time period (Tomback, 1982; Lorenz, 2008; V. Kovalenko, personal observation).

### 2.2 Site selection and sampling

We established 30 whitebark pine inventory sites throughout subalpine forests in GNP with 2–4 sites per drainage (mean = 3), each separated by a minimum of 300 m. To increase logistical efficiency and align with previous research on whitebark pine in GNP, we chose to study stands located near previous whitebark pine research and monitoring locations (Asebrook & Hintz, 2019; Kendall et al., 1996; Smith et al., 2008) that were accessible in 1 day. Upon first arrival at each of these stands, we established site centers by following a random compass bearing until reaching a clearing with a standing dead tree or rock that was positioned in a way to allow point count surveys and for attaching an acoustic recorder (minimum distance of 5 m to the nearest live tree to avoid obstacles to sight and sound). Stand characteristics were measured within four cardinally oriented 50 × 20 m transects originating at the center (4000 m^2^ total), including the number of whitebark pine cones per site (all cones were counted on all whitebark pines), density of live whitebark pines, densities of standing dead and dead‐down whitebark pines, and incidence and severity of white pine blister rust (Kovalenko, 2023; Six & Newcomb, 2005). At the center of each site, one researcher conducted a point count survey during each site visit. Depending on weather and logistics, between 0 and 3 point count surveys occurred at each site (mean = 2.51, median = 3, SD = 0.72) during each of the 2020, 2021, and 2022 nutcracker harvest seasons (late July through early October). Every Clark's nutcracker within 100 m of the observer (area of survey = 0.0314 km^2^) determined to be using the stand (foraging, caching, or perching) was noted, along with its distance and direction from the observer (Schaming, 2015). Birds simply flying through the stand were not counted. We avoided double‐counting individuals by removing birds already recorded from consideration (McLaren, 2022). Each point count lasted 10 min and was preceded by a 3‐min quiet period to allow for bird settling time. Survey‐level conditions were recorded, including date, time, and wind strength. Our study area size, defined as the area contained within the convex hull of all study points with a 100 m buffer, was approximately 1468 km^2^.

In addition, 20 of the 30 sites were equipped with automated acoustic recording units (ARUs; Cornell Swift Recorder), which recorded all environmental sounds between 0 and 4 kHz within an approximate radius of 100 m of the point count location (same approximate detection radius as the point count surveys). We determined this detection radius by imitating nutcracker vocalizations at a set of known distances. This detection radius was approximate, as detection from ARUs varies with weather conditions and vegetation structure. The 0–4 kHz sampling bandwidth clearly captured nutcrackers' relatively low‐frequency vocalizations (between 0 and 7 kHz with most amplitude between 0 and 4 kHz) while maximizing memory storage capacity and battery life. The ARUs recorded from 0600 to 1300 and from 1700 to 2100 daily. Recording occurred primarily in August and September (66% of all recordings) to maximize harvest season detection. Within a given year, each site had on average 64 recording days (SD: 12.5, range: 36–93).

### 2.3 Clark's nutcracker occupancy model

We fit a spatially explicit multi‐season occupancy model (Doser et al., 2022) using the point count observations to estimate Clark's nutcracker occupancy and its relation to whitebark pine characteristics. Occupancy models have two explicit components in which the true ecological process (i.e., occupancy probability) is modeled separately from an observation process that accounts for imperfect detection (i.e., false negatives) of the species of interest (MacKenzie et al., 2002; Tyre et al., 2003). The definition of “occupancy” in an occupancy model for a mobile species depends on the method of sampling, size of the sampling units, and timing of the repeat surveys (Latif et al., 2015; Steenweg et al., 2018). Following Steenweg et al. (2018), here we defined “occupancy” as the probability that the area sampled in a point count survey site intersected the home range of at least one individual Clark's nutcracker. We defined occupancy in this manner because the area sampled in a point count survey (0.0314 km^2^) was far smaller than the harvest season home range sizes of three individuals in GNP obtained via satellite tags (Appendix S1). More specifically, the home range sizes (i.e., 95% distribution of area used by birds during the harvest season) for each of the three birds were 47.30, 35.39, and 13.10 km^2^, while the core areas used (i.e., 50% distribution area) were 10.09, 8.29, and 3.24 km^2^, respectively. Given our definition of occupancy and the extensive movement of Clark's nutcrackers throughout their home ranges, we defined “detection probability” as the product of availability (i.e., the probability that an individual was present in the survey area and was in view of the observer and/or vocalizing) and perceptibility (i.e., the probability that an available individual was detected by the observer; Amundson et al., 2014).

Let *z*_*j,t*_ denote the true occupancy state (1 or 0) of Clark's nutcracker at site *j* during year *t*, where *j* = 1, …, 30 and *t* = 1, 2, 3. We modeled *z*_*j,t*_ following

(1) $$z_{j,t}∼\text{Bernoulli}\left(\psi_{j,t}\right)$$

where *ψ*_*j,t*_ was the probability of nutcracker occupancy at site *j* in year *t*. We modeled *ψ*_*j,t*_ as a function of whitebark pine characteristics and a linear effect of year. Because home ranges of individual Clark's nutcrackers were large enough to include more than one survey point, we included a spatial random effect in our model for occupancy probability to account for likely correlation in occupancy probabilities across nearby point count locations. More specifically, we had

(2) $$\begin{array}{c} \text{logit}\;\left(\psi_{j,t}\right)=\beta_{0}+\beta_{1}\cdot {\text{YEAR}}_{t}+\beta_{2}\cdot {\text{CONE}}_{j,t}+\beta_{3}\cdot {\mathrm{BA}}_{j}\\ \;+\beta_{4}\cdot {\mathrm{DBH}}_{j}+\beta_{5}\cdot {\text{INFECTED}}_{j,t}+w_{j}, \end{array}$$

where *β*_0_ is an intercept, and *β*_1_, *β*_2_, *β*_3_, *β*_4_, and *β*_5_ represent linear effects of year (YEAR_*t*_), whitebark pine cone density (CONE_*j,t*_), live basal area of whitebark pine (BA_*j*_), average live diameter at breast height (DBH_*j*_), and the proportion of trees infected with white pine blister rust (INFECTED_*j,t*_), respectively. We only used predictor variables with Pearson's correlation coefficients less than 0.7. w_*j*_ is a spatial random effect that accounted for residual spatial autocorrelation in occupancy probability while accounting for the effects of whitebark pine on nutcracker occupancy. We modeled w_*j*_ using a Nearest Neighbor Gaussian Process (NNGP; Datta et al., 2016) with 15 neighbors and an exponential correlation function. The structure of the spatial random effects when using an exponential correlation model was determined by a spatial variance parameter *σ* ^2^ that controled the magnitude of spatial variation across space, and a spatial decay parameter *ϕ* that controled the range of the spatial dependence. See Doser et al. (2022) for further statistical details on the use of NNGPs in occupancy models.

Each site *j* was visited *k* = 1, …, *K*_*j,t*_ times in each cone harvest season *t* to allow for separate estimation of occupancy probability from detection probability (MacKenzie et al., 2002). For each visit *k* at site *j* during season *t*, the observed detection or non‐detection, denoted as *y*_*j,t,k*_ was modeled conditional on the true occupancy process *z*_*j,t*_ such that

(3) $$y_{j,t,k}∼\text{Bernoulli}\;\left(p_{j,t,k}\cdot z_{j,t}\right),$$

where *p*_*j,t,k*_ is the probability of detecting a nutcracker at site *j* during visit *k* in season *t*, given that it was present at the site. We modeled detection probability according to

(4) $$\begin{array}{c} \text{logit}\;\left(p_{j,t,k}\right)=\alpha_{0}+\alpha_{1}\cdot {\text{DATE}}_{j,t,k}+\alpha_{2}\cdot {{\text{DATE}}^{2}}_{j,t,k}+\alpha_{3}\cdot {\text{WIND}}_{j,t,k}\\ \;+\alpha_{4}\cdot {\text{TOTALBA}}_{j}+\alpha_{5}\cdot {\text{YEAR}}_{t}, \end{array}$$

where *α*_0_ is an intercept, *α*_1_ and *α*_2_ are linear and quadratic effects of survey date, respectively, *α*_3_ is a linear effect of wind speed, *α*_4_ is a linear effect of total (live and dead) whitebark pine basal area, and *α*_5_ is a linear effect of survey year on detection probability.

We fit the model in a Bayesian framework using Markov chain Monte Carlo (MCMC) with the stPGOcc function in the spOccupancy R package (Doser et al., 2022). We assigned vague Gaussian priors to the occupancy and detection regression coefficients with a mean of 0 and variance of 2.72. We assigned a vague inverse‐Gamma prior with shape and scale parameters set to 2 and 1, respectively, to the spatial variance parameter. Lastly, we assigned an informative uniform prior to the spatial decay parameter *ϕ* based on home range estimates of three satellite‐tagged individual nutcrackers (see Appendix S1 for details on home range calculation), as we expected spatial correlation to arise as a result of the home range size of nutcrackers being larger than some of the inter‐site distances of our 30 sites. Specifically, we specified a prior on *ϕ* such that the lower bound of the effective spatial range (i.e., the distance at which the correlation between sites is 0.05) was approximately 4.36 km, which corresponds to the maximum diameter of the core use area (i.e., 50% distribution of area used by each bird during the harvest season) across the three individuals. The upper bound of the uniform prior was set such that the upper bound of the effective spatial range was the maximum inter‐site distance (84.31 km). This informative prior ensured our model explicitly accounted for the large home range size of individual nutcrackers relative to the inter‐site distances in our data set. We assessed goodness of fit using multiple posterior predictive checks with two different fit statistics: a Freeman‐Tukey statistic and a Chi‐squared statistic. For each of the two fit statistics, we used two different approaches to grouping the data (i.e., by site or by replicate) prior to calculating the fit statistic, which is necessary when doing goodness of fit assessments for binary data (see Doser et al., 2022 for more details). We summarized the posterior predictive checks with a Bayesian *p*‐value, where values near .5 indicate adequate model fit and values less than .1 or greater than .9 suggest poor model fit (Hobbs & Hooten, 2015). We ran the model for three chains, each with 60,000 iterations with a burn‐in period of 30,000 iterations and a thinning rate of 15. We assessed model convergence using the potential scale reduction factor (i.e., Rhat; Brooks & Gelman, 1998) and effective sample size, requiring Rhat to be less than 1.1 and effective sample sizes to be greater than 200 for the model intercept parameter and greater than 400 for all other parameters.

### 2.4 Species identification in acoustic data

All audio recordings were analyzed using BirdNET Analyzer (version 1.4; Kahl et al., 2021), a highly accurate machine learning algorithm used to identify recorded bird vocalizations by species. Default settings were used, including 0.0 overlap, 1.0 sensitivity, 0.1 minimum confidence, and four threads. The algorithm was set to listen only for Clark's nutcracker vocalizations. Nutcracker vocalizations are typically loud and unique from other natural sounds and animal vocalizations, making them ideal for automated identification by a deep artificial neural network such as BirdNET. To evaluate the algorithm's performance, we listened to a 3‐min segment of acoustic recordings from each site (*n* = 20) while viewing the accompanying spectrogram in Raven Pro, an acoustic analysis program (Raven Pro 1.6.4; K. Lisa Yang Center for Conservation Bioacoustics, 2023). Each segment began 1 min before a BirdNET‐annotated nutcracker vocalization and we recorded all true and false positives, as well as false negatives. We evaluated the algorithm's performance using the metrics of precision and recall following

(5) $$\text{Precision}=\frac{\text{True Positives}}{\text{True Positives}+\text{False Positives}}$$

(6) $$\text{Recall}=\frac{\text{True Positives}}{\text{True Positives}+\text{False Negatives}}$$

### 2.5 Clark's nutcracker vocalization activity model

We fit a generalized linear mixed model (GLMM) to assess spatio‐temporal variation in Clark's nutcracker activity using the vocalization data derived from the BirdNET Analyzer as our response variable. We used a negative binomial distribution with a log link function to account for overdispersion in the vocalization data. Our response variable was the number of detected vocalizations at each site on a given day during a given year. We included season‐ and site‐level covariates of cone density, mean live whitebark pine DBH, and proportion of whitebark pines infected with white pine blister rust. Total and live whitebark pine basal areas were not included in this model because they were collinear with each other and with mean live whitebark pine DBH for this set of sites (Pearson's *r* > .7). The effect of year was included to account for additional interannual vocalization variability not accounted for by yearly variation in the covariates. We accounted for spatial autocorrelation with an unstructured random effect of group for the 10 groups of sites located near one another (Figure 1). Linear and quadratic effects of date were included to account for variation in the detection of vocalizations over time. We estimated the linear and quadratic effects of date as random slopes across the 10 groups of sites to account for potential differences in vocalization activity patterns over time across the 1468 km^2^ study area.

This model did not explicitly account for imperfect detection, nor did it assume that birds stayed in the survey radius throughout the harvest season. Thus, the estimates produced by the model were interpreted as relative vocalization activity, not as true measures of activity or abundance. This use of detected vocalization activity rate as a measure of relative bird activity is a common approach for analyzing ARU data (Pérez‐Granados & Traba, 2021). Further, we included random slopes for the linear and quadratic effect of date and a random intercept of group to account for potential variation in the detection of vocalizations over time. Analogous approaches that do not explicitly account for imperfect detection are often used to model patterns in relative abundance over space/time (e.g., Kéry & Royle, 2021; Sauer & Link, 2011).

We fit the model in a Bayesian framework using Markov chain Monte Carlo (MCMC) sampling with the spAbundance R package (Doser et al., 2023). We assigned vague Gaussian priors to all regression coefficients, a uniform prior to the negative binomial overdispersion parameter, and an inverse‐Gamma prior for the group‐level random effect variances. We ran the model for three chains, each with 60,000 iterations with a burn‐in period of 30,000 iterations and a thinning rate of 20. Convergence assessment followed the same approach as the occupancy model analysis. Goodness of fit for the vocalization activity rate model was evaluated by posterior predictive checks using Freeman‐Tukey and Chi‐squared fit statistics and by calculating the 95% coverage rate of the daily vocalization values (Gelman et al., 2013).

To evaluate whether the parallel occupancy and vocalization rate models agreed on the importance of drivers of nutcracker occupancy/activity, we compared the predictors' effect distributions and their probabilities of being different than zero. We also visually assessed how the models predicted their respective response variable across sites.

## 3 RESULTS

### 3.1 Occupancy model with point count data

**FIGURE 2:**
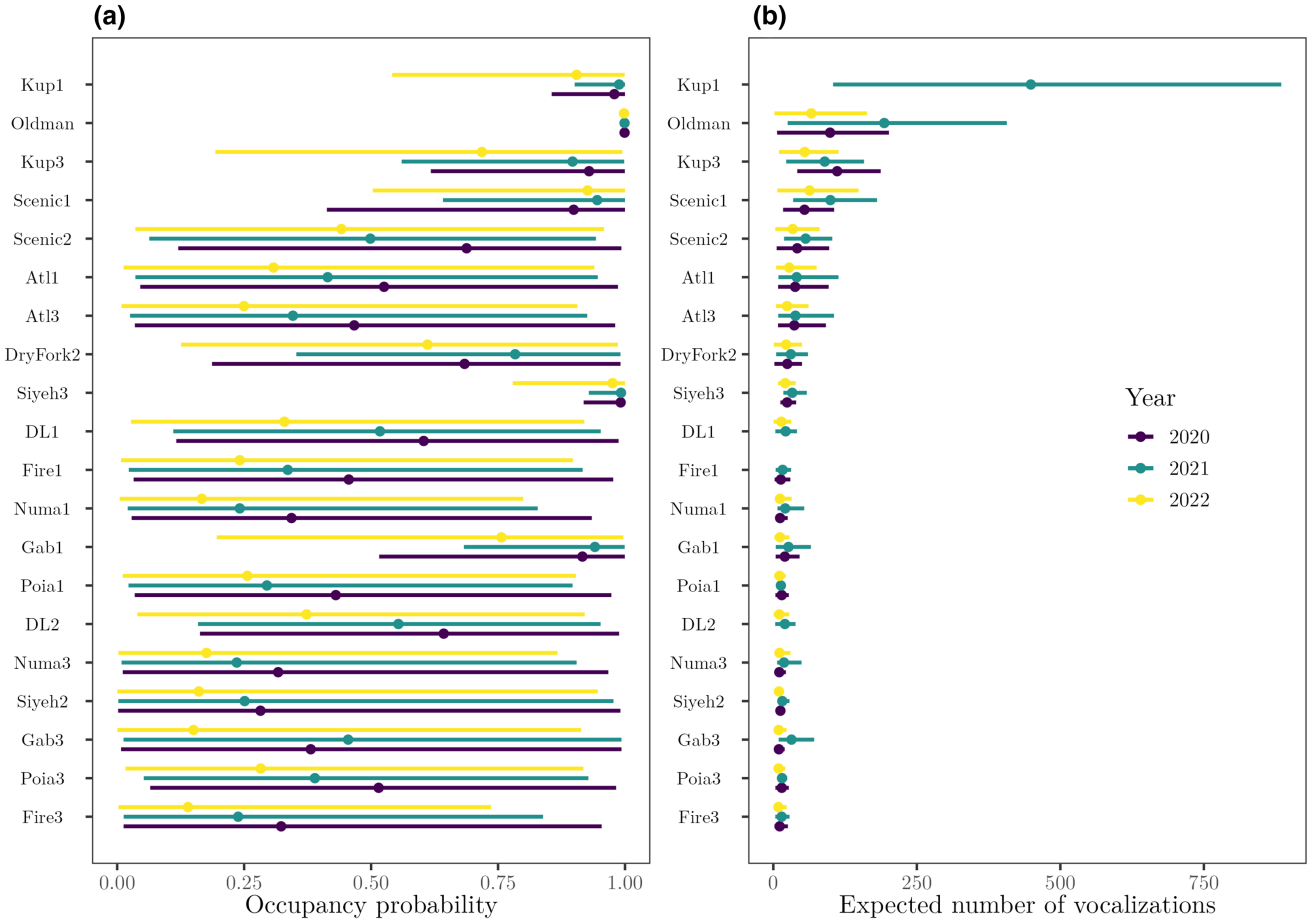
Occupancy probability (a) and expected relative vocalization activity (b) of *Nucifraga columbiana* by site and year. Only the 20 sites with acoustic data are included. Points indicate occupancy/vocalization activity means while horizontal lines indicate 95% credible intervals.

The mean number of Clark's nutcrackers detected was 0.257 detections per survey (median = 0; range = 0, 6 detections). The earliest survey of the three seasons occurred on July 8th, 2021, while the latest occurred on October 16th, 2022. The spatial multi‐season occupancy model revealed clear variation in nutcracker occupancy probability across space and time (Figure 2a). Occupancy probability was moderately lower in 2022 relative to 2020 and 2021, which generally showed similar occupancy probability on average across sites.

**FIGURE 3:**
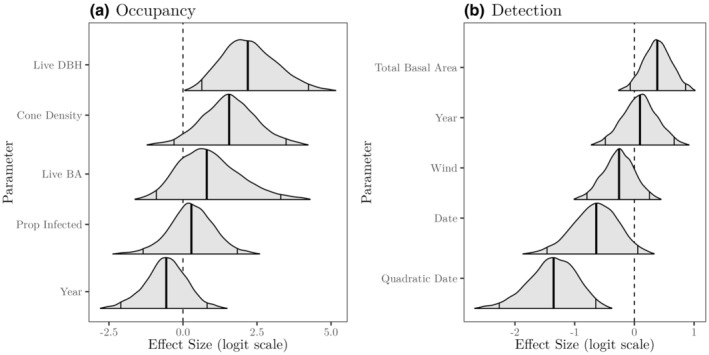
Effect sizes of (a) occupancy and (b) detection covariates in the *Nucifraga columbiana* spatial occupancy model. Density curves show covariate effect posterior distributions, bold vertical lines represent the posterior median estimates, and the non‐bold vertical lines represent the 95% credible intervals.

Mean DBH of live whitebark pines (mean = 8; median = 4; range = 0, 66 cm) and site cone density (mean = 72; median = 20; range = 0, 526 cones per site) were the two most important predictors of nutcracker occupancy (Figures 3a and 5a,b), which both had strongly supported positive relationships with nutcracker occupancy probability. There was substantial support for a positive effect of mean DBH (99.8% probability of a positive effect; 95% CI 0.64 to 4.24) and cone density (95.3% probability of a positive effect; 95% CI −0.30 to 3.48). We found marginal support for a positive effect of live basal area (mean = 17; median = 4; range = 0, 149 m^2^/ha), with a 79.9% probability of a positive effect (95% CI −0.90 to 3.30). The proportion of whitebark pine with blister rust infection (mean = 0.73; median = 0.80; range = 0.13, 1.00) did not have a discernable effect on occupancy, with an estimated effect size of 0.28 and its posterior distribution straddling zero (66.1% probability of a positive effect; 95% CI −1.34 to 1.84).

The primary predictor of Clark's nutcracker detection probability was the date of survey, with a moderately negative linear effect (96.3% probability of a negative effect; 95% CI −1.46 to 0.06; Figure 3b) and a strongly negative quadratic effect (100% probability of a negative effect; 95% CI −2.26 to −0.65; Figure 3b). These effects correspond to a peak of detection probability in mid‐August (Figure S3). Total whitebark pine basal area (live and standing dead trees; mean = 90; median = 12; range = 0, 699 m^2^/ha) and wind speed had marginally positive and negative effects on nutcracker detection probability, respectively, while year had no discernable effect (Figure 3b).

Estimates of the effective spatial range and spatial variance of the spatial random effects revealed substantial spatial autocorrelation in occupancy probability across the study region. The mean spatial variance was 1.00 (95% CI 0.18 to 4.44), while the mean effective spatial range was 7.57 km (95% CI 4.44 to 45.5 km). Notably, the uncertainty in both estimates is large as a result of the fairly small number of spatial locations used to fit the model (30). Importantly, the effective spatial range of 7.57 km is much smaller than the maximum distance between points in the study area (84.31 km).

Posterior predictive checks revealed the spatial multi‐season occupancy model adequately fit the data when using multiple test statistics and grouping the data by site or sampling events, with all Bayesian *p*‐values between .44 and .77. The model showed adequate convergence, with all R‐hat values less than 1.02 and effective sample sizes greater than 2000 for all model parameters.

### 3.2 BirdNET analyzer vocalization annotation performance

BirdNET Analyzer detected a total of 139,461 nutcracker vocalizations over the study with a mean of 39.0 daily detections (SD = 99.8), a median of 4, and a range of 0–1176. The algorithm correctly labeled all the nutcracker vocalizations it detected with a precision of 1, or 100% (i.e., there were no false positives). The algorithm's recall was 0.48, indicating that roughly half of the nutcracker vocalizations that were detectable to the human ear or visible on the spectrogram went undetected by BirdNET.

### 3.3 Clark's nutcracker vocalization activity model

Clark's nutcracker vocalization activity varied by site and year (Figure 2b). Overall, vocalization activity was highest in the 2021 harvest season and lowest in 2022. All sites except one were predicted to have the most vocalizations in 2021.

**FIGURE 4:**
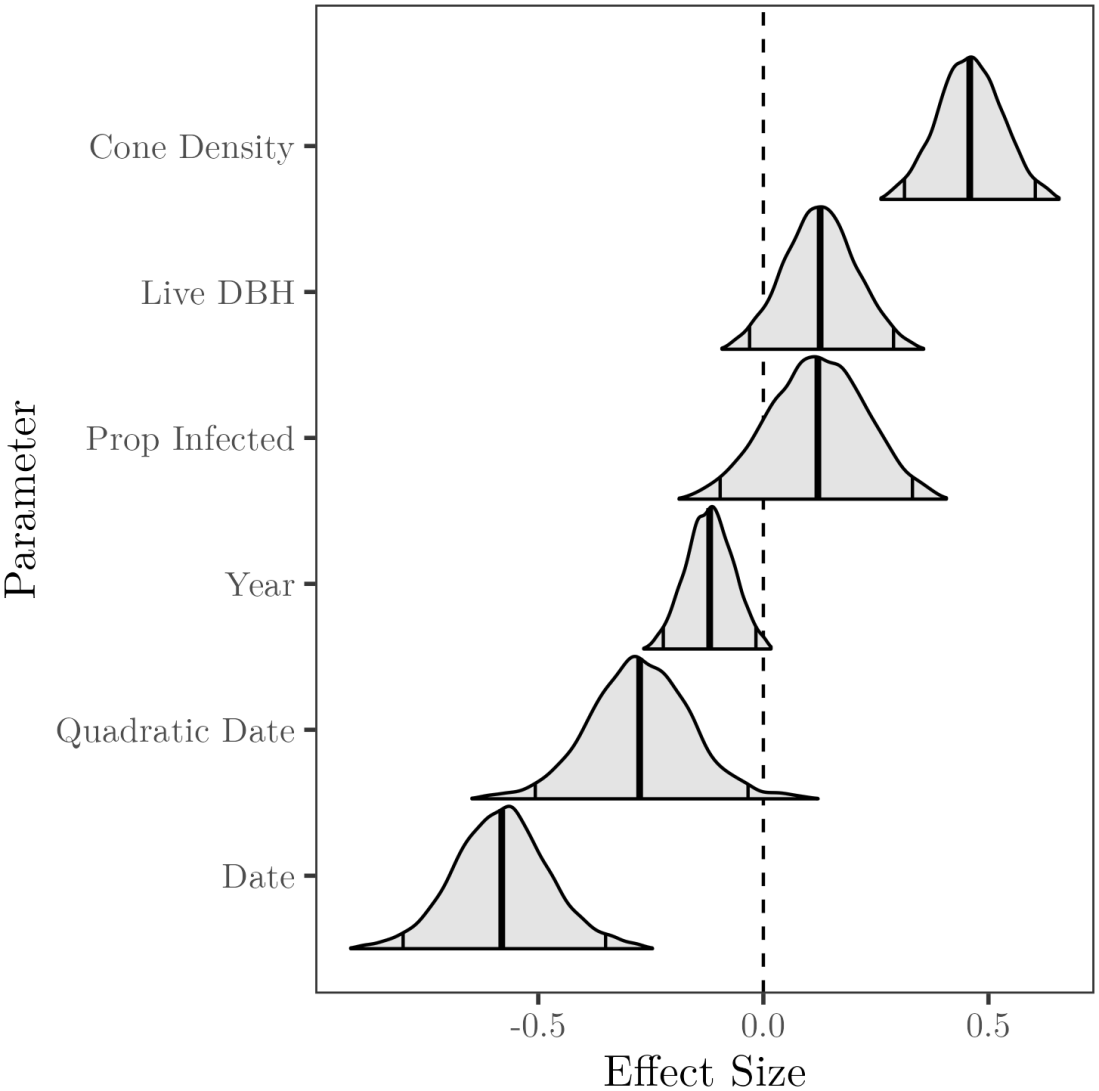
Effect sizes of relative vocalization activity covariates in the *Nucifraga columbiana* generalized linear mixed model. Curves show covariate effect posterior distributions, bold vertical lines represent the posterior median estimates, and the non‐bold vertical lines represent the 95% credible intervals.

The most important habitat variables in predicting Clark's nutcracker vocalization activity were cone density, live whitebark pine DBH, and the proportion of whitebark pine infected with white pine blister rust (Figure 4). Cone density had the largest effect size with a 100% probability of a positive effect (95% CI 0.31 to 0.60). Live whitebark pine DBH also positively influenced vocalization activity with a 94.0% probability of a positive effect (95% CI −0.03 to 0.29). The proportion of blister rust‐infected whitebark pine had a marginal positive influence on vocalization activity with an 86.3% probability of a positive effect (95% CI −0.10 to 0.33). The temporal survey‐level effects of date, its quadratic, and year all had negative influences on nutcracker vocalization activity rate.

All R‐hat values were less than 1.02, the effective sample size for the intercept was 217, and all other parameters had effective sample sizes greater than 400. Bayesian *p*‐values for the vocalization activity rate model had a mean of 0.97 between the Freeman‐Tukey and Chi‐squared tests, indicating that the model vocalization activity rate estimates had greater variability than the true site vocalization activity rates. A graphical posterior predictive check comparing the sum of true vocalizations across sites to the sum of the model‐estimated vocalizations showed reasonable correspondence (Figure S4), indicating that the model adequately estimated the spatial variability in the data. Additionally, the 95% coverage rate (Gelman et al., 2013) was examined to determine the proportion of data points for which the observed data value was within the 95% CI of the replicate data generated from the model. The target value for this test is 0.95. A model that inadequately accounts for variability will yield a value substantially lower than 0.95, whereas a higher value means there is more variability in the model estimates than in the true data. The resulting value for the vocalization activity rate model was 0.978, indicating that the model adequately fit the vocalization data for the primary purpose of assessing covariate effects.

## 4 DISCUSSION

Understanding the drivers of Clark's nutcracker occupancy is crucial to effective whitebark pine recovery management plans. The tree's reliance on the bird for seed dispersal requires land managers to understand the factors that are most important to maintaining a nutcracker population in a given area to propagate the pines. In this study, we found that the key whitebark pine stand characteristics driving nutcracker occupancy and relative activity in Glacier National Park are cone production and live tree diameter. The parallel use of point count surveys within an occupancy modeling framework together with ARUs, automated vocalization detection algorithms, and GLMMs provided multiple lines of evidence to assess the relationship between Clark's nutcracker and whitebark pine forest characteristics.

**FIGURE 5:**
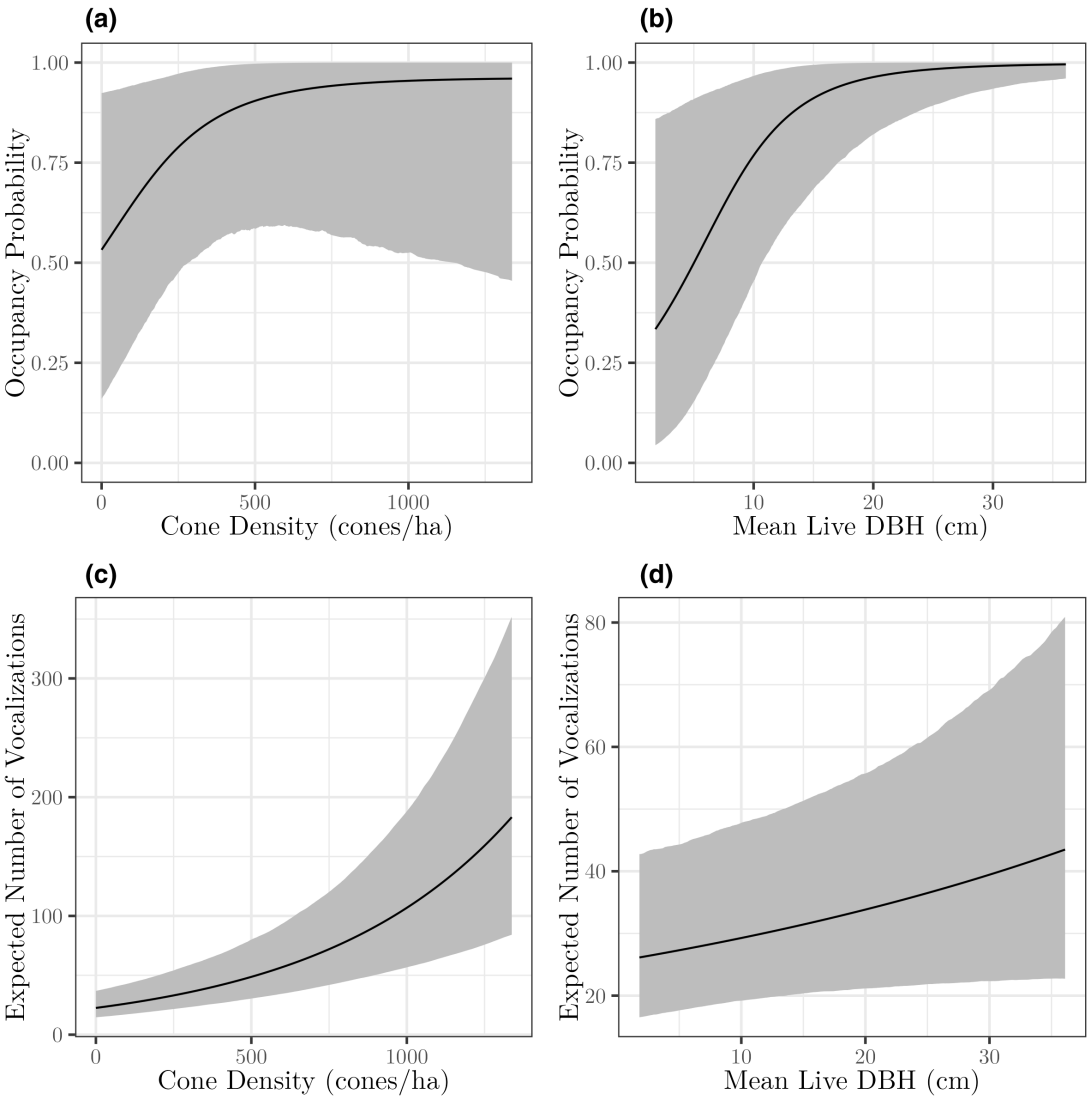
Estimated relationships between cone density and mean live whitebark pine diameter at breast height (DBH) with occupancy probability (a, b) and relative vocalization activity (c, d). Effects are shown when all other model covariates are set to their average values. Black lines represent posterior means and gray regions represent the 95% credible intervals.

Cone density was an important determinant of Clark's nutcracker occupancy and relative activity of whitebark pine stands. The multi‐season spatial occupancy model predicted that nutcrackers had a roughly 50% probability of occurring in sites without cones, increasing to a 75% probability with 250 cones per ha, and reaching nearly 100% at 1000 cones per ha (Figure 5a). The GLMM using the vocalization data showed similar results, with higher vocalization activity at higher cone densities, as well as lower, but continued nutcracker vocalization activity at low cone densities (Figure 5c). These findings are supported by Maier (2012), who found that nutcracker visitation continued at low cone density whitebark pine stands in GNP.

Mean live whitebark pine DBH was a strong predictor of Clark's nutcracker occupancy and vocalization activity rate (Figure 5b,d). Similarly, increasing whitebark pine live basal area had a positive influence on nutcracker occupancy in this study, although the effect was marginal and only interpreted in the occupancy model as it was highly correlated with other stand metrics for the set of ARU sites. Mean live whitebark pine DBH was most important in the occupancy model and second most important in the vocalization activity rate model. This suggests that while nutcrackers may be attracted to denser whitebark pine stands, they are more associated with the presence of large trees, which is likely because large trees produce more cones. Together with the strong positive effect of cone density on both occupancy probability and vocalization activity rate, these results indicate that the presence of large‐diameter whitebark pine trees and high cone densities are key factors in predicting Clark's nutcracker use of whitebark pine stands.

Analyses of traditional point count survey data with occupancy models and analyses of vocalization data from ARUs and machine learning algorithms largely provided similar findings regarding the drivers of Clark's nutcracker habitat use over space and time. They both predicted positive effects for cone density and mean live whitebark pine DBH and deemed these effects to be important to nutcracker habitat use. The proportion of whitebark pines infected with white pine blister rust appeared to have a slightly positive influence on nutcracker occupancy and vocalization activity rate; however, its effect had the lowest magnitude of any site covariate in either model. While it is unclear whether or how the level of rust incidence influences nutcracker foraging decisions, it is evident from these models that the size of live whitebark pines and the quantity of cones produced in the stand are significantly more important determinants of nutcracker presence/activity. We also note that the small effect of white pine blister rust is difficult to interpret, as it may be confounded with the increased probability of infection in larger whitebark pines (Smith et al., 2001; Thoma et al., 2019).

Here we used daily vocalization activity rate derived from ARU recordings in a GLMM to quantify relationships between relative vocalization activity and whitebark pine characteristics. Vocalization activity rate is a common metric derived from passive acoustic monitoring surveys to understand patterns in bird activity (Pérez‐Granados & Traba, 2021; Sugai et al., 2019). This metric is sensitive to variation in vocalization rates over time, which we accounted for in our GLMM using random linear and quadratic effects of the day of survey. Further, while vocalization rate may be positively correlated with abundance, this relationship is likely complex and nonlinear, making the link between abundance and vocalization activity unclear. For example, a site with a large number of vocalizations may be indicative of multiple individuals, or a single individual vocalizing multiple times. However, a higher number of vocalizations at one site on average across multiple recordings obtained over time likely indicates that site has better characteristics for the species of interest compared to a site with fewer vocalizations, as it would correspond to either a single individual spending large amounts of time within the area sampled by the ARU, or multiple individuals using that area.

Augmenting traditional point count surveys with passive acoustic monitoring allowed us to obtain multiple lines of evidence for quantifying relationships between Clark's nutcrackers and whitebark pine forests. More specifically, we used state‐of‐the‐art machine learning tools (i.e., BirdNet; Kahl et al., 2021) and GLMMs to extract a measure of relative vocalization activity from the acoustic recordings, which provided us the ability to quantify relationships between relative activity and whitebark pine characteristics, in addition to the traditional point count surveys that quantified relationships between Clark's nutcracker occupancy and whitebark pine characteristics. We found both approaches provided similar findings, highlighting the importance of high cone density and the presence of large‐diameter whitebark pine trees are most associated with high activity/occupancy of Clark's nutcracker. This use of two distinct data streams to identify important whitebark pine forest characteristics for Clark's nutcracker gives us increased confidence in the conclusions drawn from either individual data source.

An alternative approach to performing separate analyses for the two data sources would seek to fit a single, integrated model using the two data sources to estimate occupancy and/or abundance (e.g., Doser et al., 2021; Kéry & Royle, 2021; Van Wilgenburg et al., 2017). In initial stages of this analysis, we found the integrated model developed by Doser et al. (2021) led to extremely large and unreasonable estimates of relative abundance, which was primarily related to substantial violations of the closure assumption that the N‐mixture‐based integrated model relies upon. We thus pursued separate analyses using the two distinct data streams. The development of integrated approaches that can reliably estimate activity and/or abundance without population closure assumptions would be valuable for studying highly mobile species like Clark's nutcracker.

## 5 CONCLUSIONS

The decline of whitebark pine in GNP and in many other parts of the species' range has far‐reaching implications for the treeline and subalpine communities it supports as well as on Clark's nutcracker distribution and survival (Tomback & Achuff, 2010; Tomback et al., 2016). Our results suggest that while Clark's nutcrackers spend more time in stands that contain more cones and larger whitebark pines, they continue to visit stands with fewer cones and large trees. Given nutcrackers' large‐scale movements and home ranges, it is not unexpected that they occur across the spectrum of whitebark pine stand health and productivity. This is encouraging as the current restoration strategies rely on the assumption that nutcrackers will return once the landscape has been restored with healthy, rust‐resistant trees. Our results may help managers to prioritize stands where restoration is more likely to increase Clark's nutcracker visitation and natural whitebark pine regeneration.

## AUTHOR CONTRIBUTIONS

**Vladimir Kovalenko:** Conceptualization (lead); data curation (lead); formal analysis (equal); investigation (equal); methodology (equal); software (equal); visualization (equal); writing – original draft (lead); writing – review and editing (lead). **Jeffrey W. Doser:** Formal analysis (equal); methodology (equal); software (equal); visualization (equal); writing – original draft (supporting); writing – review and editing (equal). **Lisa J. Bate:** Conceptualization (equal); funding acquisition (equal); methodology (equal); writing – review and editing (equal). **Diana L. Six:** Project administration (equal); supervision (equal); writing – review and editing (equal).

## FUNDING INFORMATION

Funding for this research was generously provided by the Natural Resource Stewardship and Science Directorate (Project #230375) and the Glacier National Park Conservancy.

## CONFLICT OF INTEREST STATEMENT

The authors have no known conflicts of interest.

## Supporting information


Appendix S1.

## Data Availability

All data and code used in the analyses may be found at https://github.com/vladkov88/nutcracker.
