# Supplementary material for: Paired acoustic recordings and point count surveys reveal Clark's nutcracker and whitebark pine associations across Glacier National Park
Source: Ecol Evol. 2024 Jan 24;14(1):e10867. doi: 10.1002/ece3.10867 (PMC10808773; doi:10.1002/ece3.10867)
Supplement: Supplementary file 1 — Appendix S1. [file ECE3-14-e10867-s001.pdf]

Supplement

Clark's Nutcracker Satellite Tracking and Home Range Estimation

The original objectives of this study included elucidating nutcracker migration strategy. To that end, 8 nutcrackers were captured and equipped with backpack solar Platform Transmitter Terminal (Solar 5g PTT, Microwave Telemetry, Inc.) and tracked via satellite (Argos; CLS America). All birds were handled according to Institutional Animal Care and Use Committee guidelines (National Institutes of Health, 1992). Due to performance issues with the transmitters (likely insufficient sunlight on the solar panels in winter) as well as two confirmed bird mortalities and only three birds transmitted enough location data to calculate home range (HR) estimates during the harvest seasons and one of them left the GNP ecosystem in fall of 2022.

Clark's nutcracker HRs in 2021 and 2022 were estimated for three satellite-tagged individuals using the Continuous-Time Movement Model package in R (CTMM; Fleming & Calabrese, 2022). The methods used in CTMM are an improvement over traditional kernel density estimation (KDE), which can lead to biased area estimates and confidence intervals. CTMM introduces autocorrelation kernel density estimation (AKDE) which accounts for the fact that temporally adjacent animal locations are not independent of each other (Fleming & Calabrese, 2017). Model selection is performed through visual parameter assignment based on the frequency and variance of the location data used (Fleming et al., 2015; Calabrese et al., 2016). An advantage of CTMM is its ability to accurately estimate HR with small sample sizes, which made it possible to estimate HRs for three birds over two harvest seasons (Fleming & Calabrese, 2017; Fleming et al., 2019). To mitigate bias in HR estimation, only location data with an error radius of less than 500 m was used (Christin et al., 2015; CLS, 2016). We calculated the core use areas and home ranges as the 50% and 95% distributions of area used by

the birds during the harvest season, respectively, as well as the respective 95% confidence intervals. These estimates were compared across years and whitebark pine cone crops.

Figure S1 shows the core area and home range estimates for each satellite-tracked bird in each year, while Figure S2 provides maps of home ranges by individual and year. All three birds used the same areas in both years. The mean size of core areas used by the three individuals over both harvest seasons was 7.2 km^2 (SD = 4.2 km^2), while the mean of their home ranges was 32.0 km^2 (SD = 18.6 km^2). There did not appear to be any systematic pattern in year-to-year size of core use area and home range for individual birds. One bird's area of use increased, another bird's remained roughly the same, and the third's decreased from 2021 to 2022. Annual core use area and home range sizes were weakly correlated to cone crops with Pearson's correlation coefficients of -0.20 and -0.18, respectively. Importantly, most area use estimates had high uncertainty due to low sample sizes. For example, in 2022, bird 450 only transmitted 4 high-quality points.

Due to the small samples of satellite-tagged birds and high-quality location datapoints, the estimates of Clark's nutcracker harvest season HRs were highly uncertain (Figure 2). Thus, it was not possible to infer how nutcracker harvest season HRs changed in relation to this study's sole annually variable habitat metric – cone density.

Schaming (2016) found that nutcracker breeding season HR was smaller following a high whitebark cone crop than a low one, and breeding nutcrackers had smaller HRs than non-breeders (Schaming, 2016). This may be due to an abundance of cached cones near the birds' core use areas and nesting territories. In this study, one bird's (445) core use area was significantly smaller in a relatively high cone year, 2021, than a low cone year, 2022. However, for a different individual (451), the opposite was true. Bird 451's core use area shrank

significantly in 2022, suggesting it may have bred; however, this could not be confirmed. The third individual's (450) core use area and HR did not change significantly and had the widest confidence intervals. In the fall of 2022, bird 445 began transmitting locations outside of GNP. This bird left the GNP ecosystem in early October 2022 and has been transmitting from the Sawtooth and Challis National Forests to the time of writing for this paper. This is the only instance of long-distance migratory behavior that has been observed in GNP nutcrackers in this or any study.

As a result of the wide confidence intervals and lack of systematic response between years and among individuals, conclusions about how the whitebark pine cone crop affected nutcracker harvest season HRs or migration could not be drawn. Nonetheless, an important finding of this analysis was that given these animals' extensive HRs, latent state abundance models with site-level population closure assumptions cannot be reliably fit to estimate Clark's nutcracker site abundance at this scale. These birds cover vast distances throughout the harvest seasons that are well beyond the detection radii of human observers and ARUs. As a result, abundance modeling of this species here was restricted to predicting nutcracker vocalization activity at the site level, yielding an index of relative vocalization activity among sites.

References

CLS America, 2020. *Argos user's manual*. Retrieved from: http://www. argos-system. org/manual.

Calabrese, J.M., Fleming, C.H. and Gurarie, E., 2016. ctmm: an r package for analyzing animal relocation data as a continuous-time stochastic process. *Methods in Ecology and Evolution*. Edited by R. Freckleton, 7(9), pp. 1124–1132. Available at: https://doi.org/10.1111/2041-210X.12559.

Christin, S., St-Laurent, M.-H. and Berteaux, D., 2015. Evaluation of Argos Telemetry Accuracy in the High-Arctic and Implications for the Estimation of Home-Range Size. *PLoS One*, 10(11), p. e0141999. Available at: https://doi.org/10.1371/journal.pone.0141999.

Fleming, C.H., Fagan, W.F., Mueller, T., Olson, K.A., Leimgruber, P. and Calabrese, J.M., 2015. Rigorous home range estimation with movement data: a new autocorrelated kernel density estimator. *Ecology,* 96(5), pp.1182-1188.

Fleming, C.H., Noonan, M.J., Medici, E.P. and Calabrese, J.M., 2019. Overcoming the challenge of small effective sample sizes in home-range estimation. *Methods in Ecology and Evolution*, 10(10), pp.1679-1689.

Fleming, C.H. and Calabrese, J.M., 2017. A new kernel density estimator for accurate home-range and species-range area estimation. *Methods in Ecology and Evolution*, 8(5), pp. 571–579. Available at: https://doi.org/10.1111/2041-210X.12673.

Fleming, P.A., Wentzel, J.J., Dundas, S.J., Kreplins, T.L., Craig, M.D. and Hardy, G.E.S.J., 2021. Global meta-analysis of tree decline impacts on fauna. *Biological Reviews*, 96(5), pp.1744-1768.

National Institutes of Health (US). Office for Protection from Research Risks and Applied Research Ethics National Association, 1992. *Institutional Animal Care and Use Committee Guidebook* (No. 92). US Department of Health and Human Services, Public Health Service, National Institutes of Health.

Supplementary Material Figures

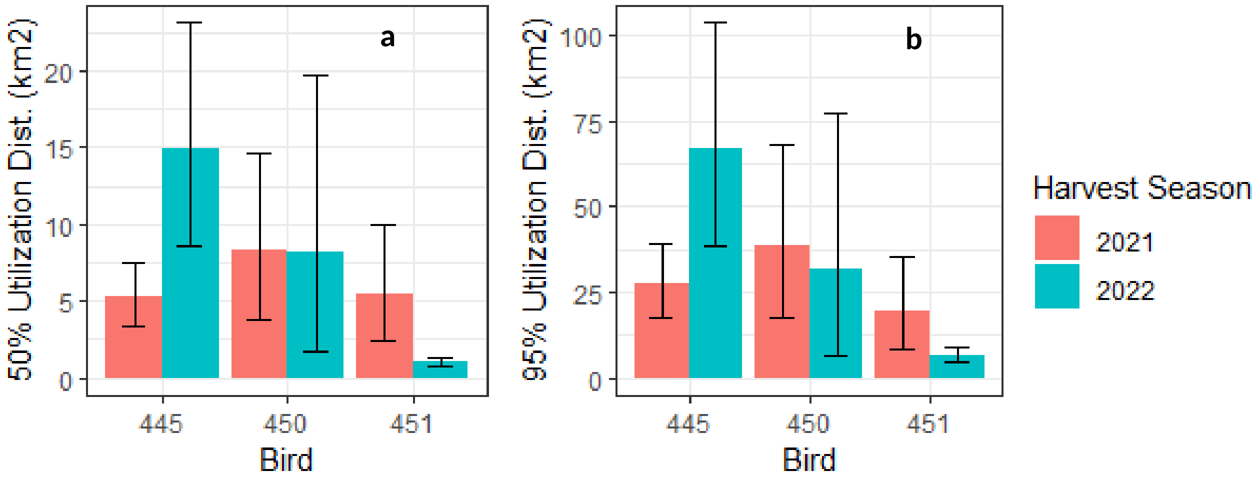

Figure S1. Clark's nutcracker home range estimates with core use areas (a) and home ranges (b) for each satellite-tracked bird (445, 450, and 451) during 2021 and 2022 cone harvest seasons (late July through September). Error bars represent the 95% confidence intervals.

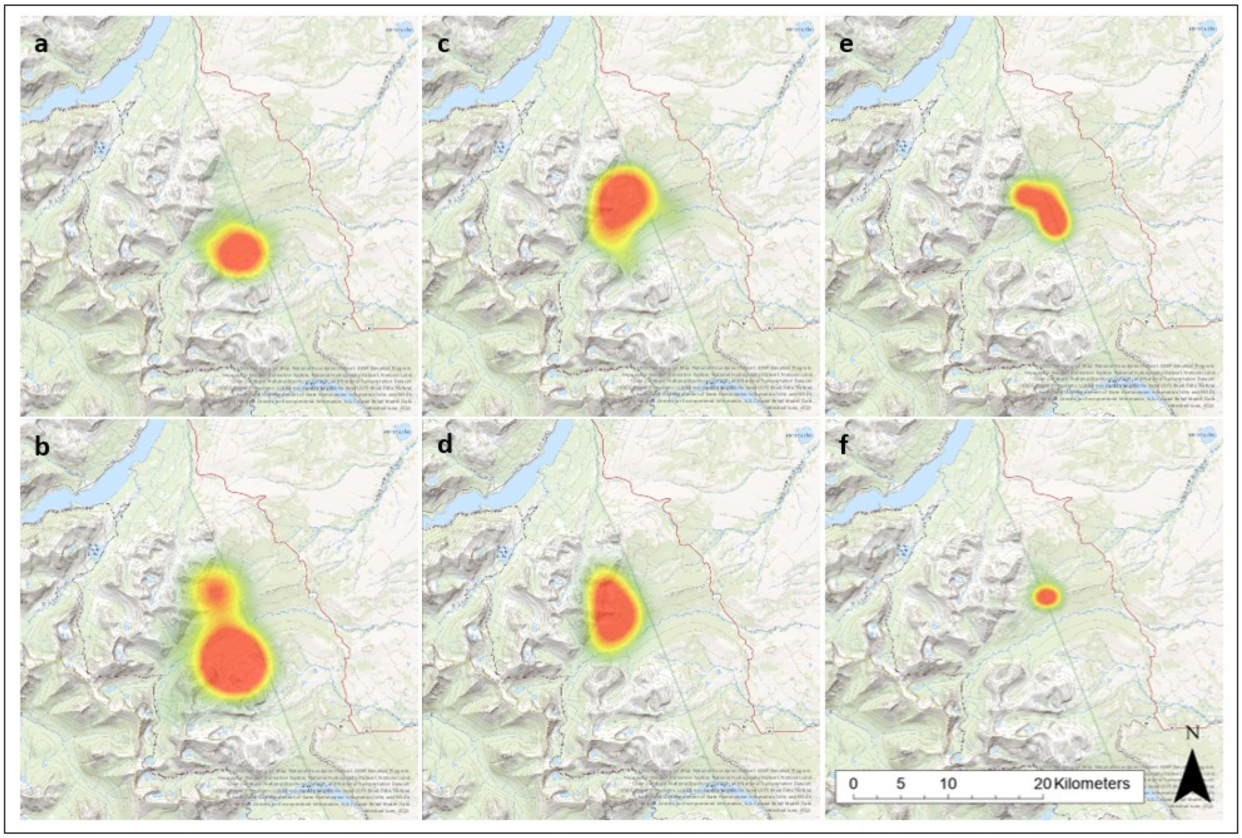

Figure S2. Maps comparing Clark's nutcracker harvest season (late July through September) home ranges. Columns separate birds while rows separate years. (a) Bird 445 in 2021, (b) bird

445 in 2022, (c) bird 450 in 2021, (d) bird 450 in 2022, (e) bird 451 in 2021, and (f) bird 451 in 2022.

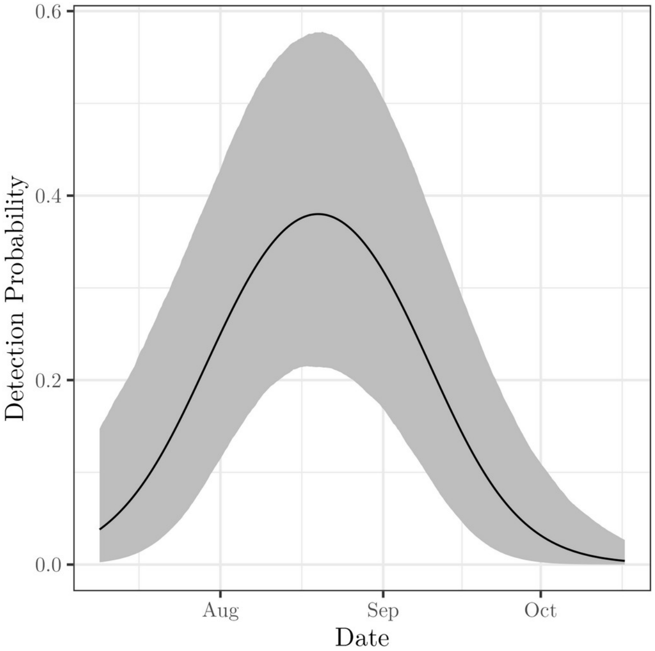

Figure S3. Relationship between Clark's Nutcracker detection probability and calendar date as estimated in the spatial occupancy model. Black line represents the posterior mean and the grey region represents the 95% credible interval.

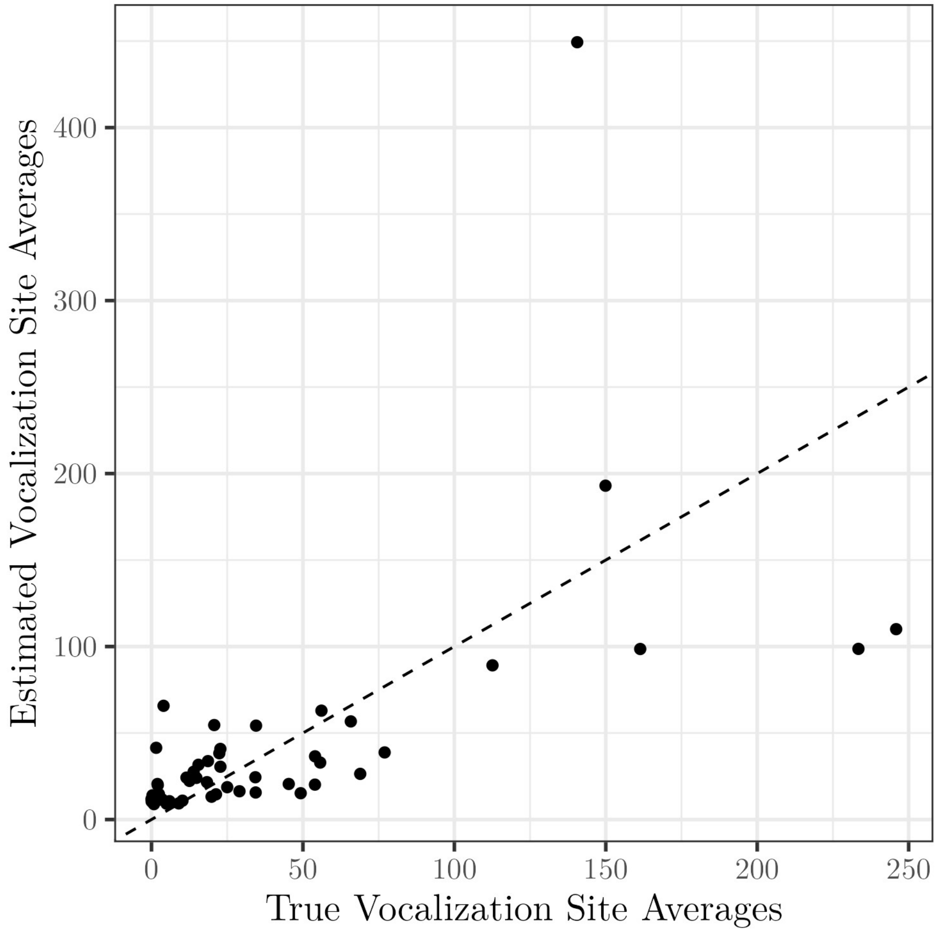

Figure S4: Scatterplot of true (x-axis) versus predicted (y-axis) mean site vocalization estimates from the generalized linear mixed model.

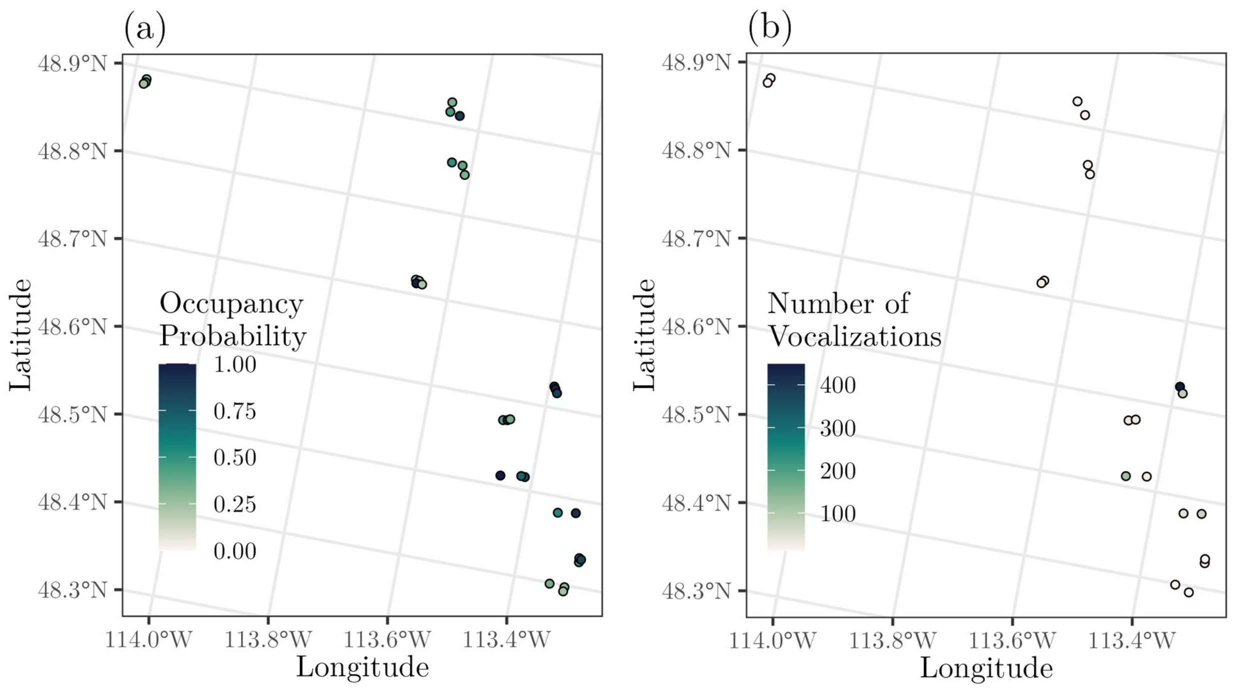

Figure S5: Mean occupancy probability (a) from a spatial occupancy model and mean number of vocalizations (b) from a generalized linear mixed model. Values represent the average value across the three years of surveys.
